# Lack of Multisensory Integration in Hemianopia: No Influence of Visual Stimuli on Aurally Guided Saccades to the Blind Hemifield

**DOI:** 10.1371/journal.pone.0122054

**Published:** 2015-04-02

**Authors:** Antonia F. Ten Brink, Tanja C. W. Nijboer, Douwe P. Bergsma, Jason J. S. Barton, Stefan Van der Stigchel

**Affiliations:** 1 Experimental Psychology, Helmholtz Institute, Utrecht University, Utrecht, The Netherlands; 2 Brain Center Rudolf Magnus Institute of Neuroscience and Centre of Excellence for Rehabilitation Medicine, University Medical Centre Utrecht and Rehabilitation Centre De Hoogstraat, Utrecht, The Netherlands; 3 University Medical Centre St. Radboud, department of Cognitive Neuroscience, Nijmegen, The Netherlands; 4 Departments of Medicine (Neurology), and Ophthalmology and Visual Sciences, University of British Columbia, Vancouver, Canada; University of Muenster, GERMANY

## Abstract

In patients with visual hemifield defects residual visual functions may be present, a phenomenon called blindsight. The superior colliculus (SC) is part of the spared pathway that is considered to be responsible for this phenomenon. Given that the SC processes input from different modalities and is involved in the programming of saccadic eye movements, the aim of the present study was to examine whether multimodal integration can modulate oculomotor competition in the damaged hemifield. We conducted two experiments with eight patients who had visual field defects due to lesions that affected the retinogeniculate pathway but spared the retinotectal direct SC pathway. They had to make saccades to an auditory target that was presented alone or in combination with a visual stimulus. The visual stimulus could either be spatially coincident with the auditory target (possibly enhancing the auditory target signal), or spatially disparate to the auditory target (possibly competing with the auditory tar-get signal). For each patient we compared the saccade endpoint deviation in these two bi-modal conditions with the endpoint deviation in the unimodal condition (auditory target alone). In all seven hemianopic patients, saccade accuracy was affected only by visual stimuli in the intact, but not in the blind visual field. In one patient with a more limited quadrantano-pia, a facilitation effect of the spatially coincident visual stimulus was observed. We conclude that our results show that multisensory integration is infrequent in the blind field of patients with hemianopia.

## Introduction

The predominant pathway for projection of visual information is the retinogeniculostriate pathway, which projects from the retina to the lateral geniculate nucleus and then to the primary visual cortex. Damage of this pathway results in a loss of conscious vision in the hemifield that corresponds retinotopically to the damaged area, a disorder called hemianopia. Despite the lack of consciousness of visual stimuli in the blind hemifield, there may be residual visual functions in (parts of) the blind field, a phenomenon called blindsight [1–5]. The estimated incidence of blindsight is low, with most group studies finding only a few patients showing it [4,6–8]. There is debate regarding whether blindsight is due to residual function in the retinogeniculostriate pathway, or to the function of spared alternative visual pathways [9,10].

One of the alternative pathways that has been proposed to mediate blindsight is the retinotectal pathway, which projects from the retina to the superior colliculus (SC) [1,11–16]. A majority of the neurons in the SC receive information from more than one sensory area and modality, for example the occipital, auditory and somatosensory cortices [17,18]. Because of these projections, neurons in the SC process input from the visual, auditory and somatosensory systems and several forms of multisensory interaction take place [13,19–22]. Since the SC is spared in patients with hemianopia, information from the visual modality in combination with other modalities in the blind hemifield could still be processed in these patients [23–27].

Frassinetti et al. [28] investigated whether conscious perception of visual stimuli in the blind hemifield of patients with hemianopia could be improved by presenting bimodal stimuli. They observed that detection of a visual target in the blind hemifield was enhanced by adding an auditory stimulus at the same location. In another study, Leo et al. [29] asked patients with hemianopia to verbally report the location of an auditory target that was presented either alone or with a visual stimulus at various spatial disparities between the two. In the intact ipsilateral hemifield, localization of the auditory stimulus was highly influenced by the visual stimulus. When both stimuli were presented at the same location, localization accuracy was enhanced. When the stimuli were spatially disparate, localization accuracy was reduced and biased towards the visual stimulus. In the blind hemifield, the visual stimulus influenced the localization of the auditory stimulus only when the stimuli were spatially coincident. In this case, localization accuracy of the auditory stimulus was enhanced by the visual stimulus in the blind field. Presenting a visual stimulus at a different location did not affect auditory localization. These results can be explained by the assumption that the enhancement effect and the effect of visual bias depend on different neural pathways: the SC is responsible for integration of bimodal stimuli presented at the same location, whereas the geniculostriate circuits may play a key role in the effect of visual bias. Hence Leo et al. [29] argued that the enhancement effect occurs in patients with hemianopia since the SC is spared, but no visual bias occurs because the geniculostriate circuits are damaged.

Besides multimodal integration, the SC is of crucial importance in orienting behaviours, like programming saccades [18,30]. A number of studies investigated how saccadic eye movements are influenced by bimodal stimuli, in particular the interaction between visual and auditory stimuli [13,23,31–39]. Researchers demonstrated that saccade latency and saccadic accuracy can be modulated by input from both sensory modalities [32–34]. Based on neurophysiological recordings, Stein and Meredith [39] proposed three main principles regarding multimodal integration at the single cell level. First, the spatial rule states that only spatially aligned stimuli from different modalities will integrate, since integration depends on the organization of receptive fields of multisensory neurons. Second, the temporal rule states that stimuli presented without temporal disparity are enhanced most. Last, the rule of inverse effectiveness states that combining weak unimodal stimuli produce relatively more enhancement than combining strong unimodal stimuli [13,31]. Comparable effects have been demonstrated in humans [40]. Saccades made to audiovisual stimuli in close spatial and temporal proximity are faster than saccades to a unimodal stimulus [35,37,41]. Additionally, saccadic accuracy is enhanced in case of audiovisual stimuli that are spatially and temporally aligned, but if the auditory stimulus is at a different location than the visual target, saccades to the visual target are less accurate than saccades to a visual target only [35]. Following the temporal rule, the degree of influence of the distractor depends on the moment of presentation: when both stimuli are presented within a certain temporal window the enhancement is the largest [36].

The use of multisensory stimuli and a saccade paradigm to study residual visual function in patients with hemianopia is promising, as the SC is involved in both multisensory integration and programming saccades. Furthermore, saccades are more reflexive than verbal reports of target location [42], and are shown to be more sensitive to subliminal information [43]. Until now, four studies have investigated the influence of non-coincident visual distractors in the blind hemifield at saccades made to seen visual stimuli in the intact hemifield [4,8]. Regarding saccade accuracy, two of three hemianopic patients showed a shift of saccade endpoints to a visual target in the intact hemifield, when there was a simultaneous visual distractor in the blind hemifield [4]. In another study with a comparable experimental design, in two of five patients a visual distractor in the blind hemifield altered trajectories of saccades towards a seen target in the intact hemifield [8]. With respect to saccade latency, results have been inconsistent between studies. In a first study, three hemianopic patients had to make saccades to a box in the intact field as soon as they saw it brighten. Saccade latency increased in all three patients if a box in the blind hemifield also brightened within a temporal window of 50 ms [14]. However, this effect could not be replicated in another study with six patients [44]. These inconsistent results could possibly be explained by the low and variable incidence of blindsight. However, previous studies that presented both visual and auditory stimuli in the blind hemifield have observed multisensory enhancement at a group level [28,29]. The aim of the present study was to investigate whether multimodal integration in the blind visual field is a general and consistent phenomenon in patients with visual field defects. To investigate whether multimodal stimuli in the blind visual field have an influence on oculomotor competition, we tested a sample of hemianopic patients. For each patient we determined whether and how a visual stimulus in the blind hemifield influenced saccades made to an auditory stimulus in the same hemifield. Seven patients with hemianopia and one patient with quadrantanopia were asked to make a saccade to an auditory target that was presented alone or together with a visual stimulus that was or was not spatially coincident with the auditory stimulus. We assessed eye movements made towards the intact and blind hemifield, measuring saccadic accuracy and saccade latency. We expected that in both hemifields, in line with the spatial rule, the saccadic accuracy of saccades made to an auditory target would improve when a visual stimulus was spatially aligned with the auditory target. Given the rule of ‘inverse effectiveness’, combining weak unimodal stimuli produces relatively more enhancement than combining strong unimodal stimuli [13,31]. Since saccades to an auditory target are less accurate than saccades to a visual target [41], the effects of blind visual stimuli may be particularly visible in responses to auditory stimuli. When a visual stimulus is not spatially aligned with the auditory target, we expected the visual stimulus to function as a distractor. An abrupt-onset distractor captures attention automatically, even without intention of the observer, and subsequently results in a shift of saccade endpoint towards the distractor [45–49]. For eye movements in the blind hemifield, we expected that due to the reflexive nature of eye movements, saccadic accuracy to the auditory target would be reduced in case of a remotely presented visual distractor. Such a distracting effect of a remotely presented visual stimulus was not demonstrated in previous studies [28,29], perhaps due to the adopted measure (i.e. a verbal report instead of eye movements). Finally, with respect to saccade latency, we expected that the effects of multisensory integration would be to make saccades faster to an auditory target when a visual stimulus was also present at the same location.

## Experiment 1

### Materials and Methods

#### Participants

A pre-experimental sound selection task was performed by 11 patients with visual field defects. Only patients who were able to verbally localize sounds correctly on at least 75% of trials were selected for the experiment. Details of this task are described later.

**Fig 1:**
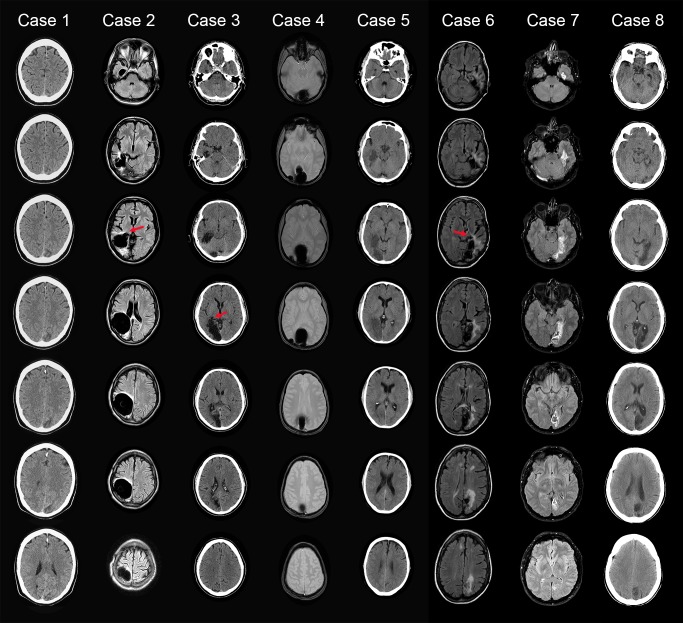
Axial images of the lesions of the participants. With CT for Cases 3, 5 and 8, MRI sequences for Cases 1, 2, 6, and 7 (FLAIR sequence) and Case 4 (SWI sequence). The arrows indicate possible LGN involvement. The left side of each slide represents the left side of the brain.

**Fig 2:**
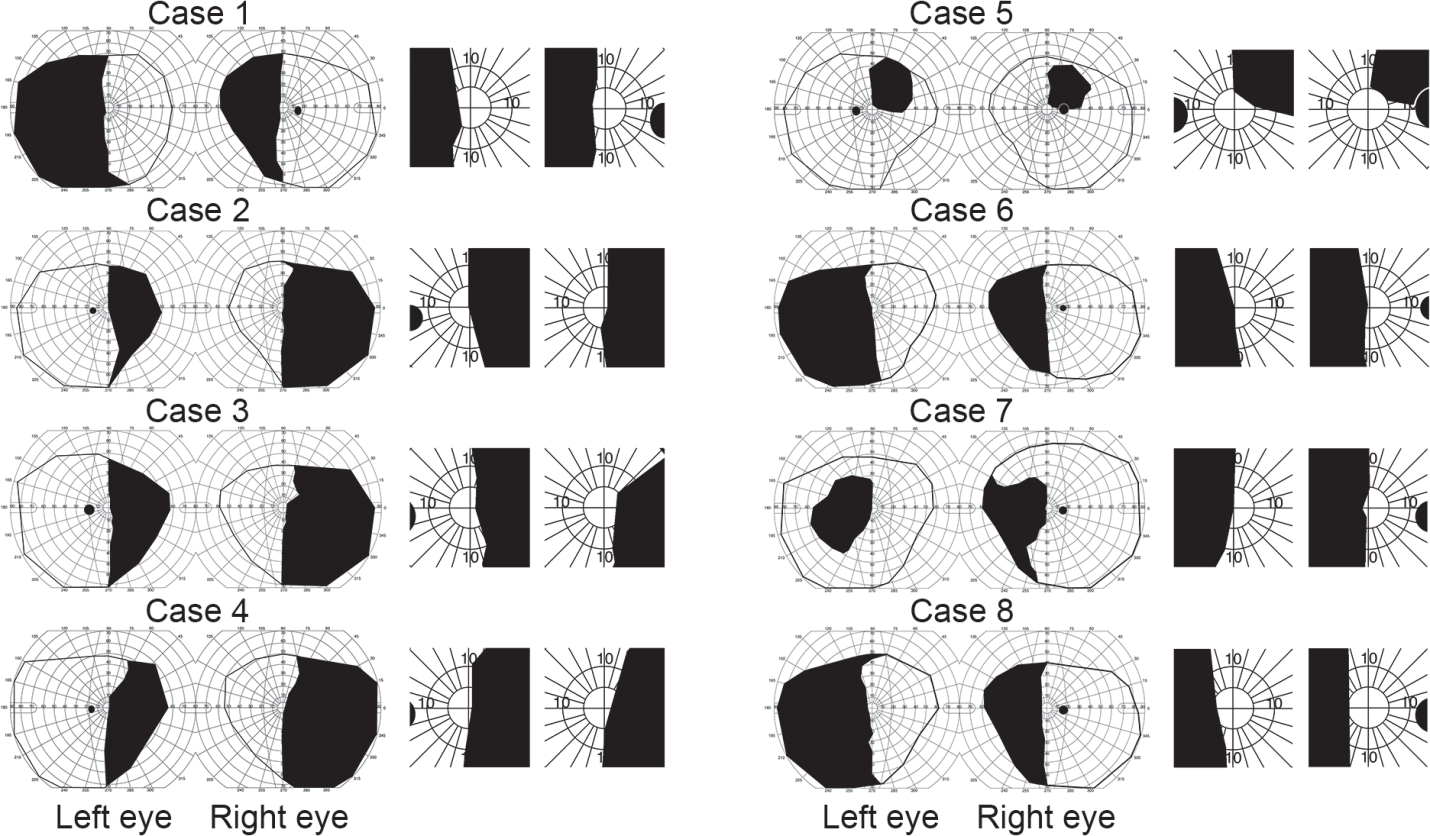
Goldmann perimetry of visual fields of all participants. Black regions indicate regions where the largest and brightest target, the V4e stimulus, was not seen. On the right side, the central zone of 10° has been magnified for each participant.

Based on the sound selection task we included five participants with lesions of the optic radiations or the striate cortex resulting in homonymous hemifield defects, due to ischemic strokes or intracerebral haemorrhages (Table 1). The lesions affected the retinogeniculostriate pathway but not the expected path of retinotectal projections. Fig. 1 shows MRI or CT images of the lesions of the participants. Results of Goldmann perimetry are shown in Fig. 2. All patients had normal or corrected-to-normal visual acuity. All participants gave written informed consent according to the standards of the Declaration of Helsinki for a protocol that was approved by the institutional review boards (METC) of the University Medical Centre Utrecht (research protocol number 07/306) and the university of Utrecht.

**Table 1:** Summary of the clinical data of the patients we studied.

Case	Age (years)	Sex	Duration (months)	Lesion information	Visual structure	Participated in experiment
1	56	M	56	medial occipital vascular malformation	striate cortex	1, 2
2	50	M	141	lateral parietal to medial temporal cyst	optic radiations, possible LGN	1
3	53	M	28	medial occipitotemporal infarct, posterior cerebral artery	optic radiations and striate cortex, possible LGN	1
4	27	F	26	medial occipital encephalomalacia	striate cortex	1, 2
5	60	M	33	medial occipitotemporal infarct, posterior cerebral artery	optic radiations and striate cortex	1
6	45	F	154	medial occipitotemporal infarct, posterior cerebral artery	optic radiations and striate cortex, gliosis extending into LGN	2
7	43	M	29	medial occipitotemporal infarct, posterior cerebral artery	striate cortex, distal optic radiations	2
8	50	F	123	medial occipitotemporal infarct, posterior cerebral artery	striate cortex, distal optic radiations	2

#### Apparatus

Eye movements were recorded by an Eyelink1000 system (SR research Ltd., Canada), an infrared video-based eye tracker that has a 1000 Hz temporal resolution and a spatial resolution of 0.01°. The participants head was stabilized using a chin rest, the distance between the screen and chin rest was 80 cm. Participants were tested with both eyes open, the left eye was monitored. The participants were seated in a sound-attenuated, dimly lit room. The visual stimuli were generated by a Toshiba TLP-T621 LCD projector (60 Hz) on a white screen; the size of the projection was 56 x 43 cm. At 0.25 cm behind the screen, eight piezoelectric loudspeakers (0.4 W, 8 Ω) were mounted on wooden stands fixed to the table surface such that auditory and visual stimuli could be spatially aligned. The experimental computer and the speakers were linked by a Fast Track Ultra 8R USB audio interface (M-Audio, Irwindale, California, United States).

#### Stimuli, procedure and design

Visual field test: To confirm the estimations of the location and size of the blind regions as measured with the Goldmann, a computerised visual field test was administered. Participants were given 120 trials, 40 without a stimulus and 80 that showed a disk of the same size and luminance as the experimental targets. The disk was shown at one of 20 possible locations in either hemifield, including the locations of the visual stimuli used in the experiment. To ensure that participants were looking at the center of the screen at the start of each trial (so that visual stimuli were correctly classified as in blind or seeing parts of the field), the experimenter at the control monitor only started the next trial by pressing the space bar when they verified that participants were fixating screen center. In each trial, participants had to report verbally whether they saw a disk.

**Fig 3:**
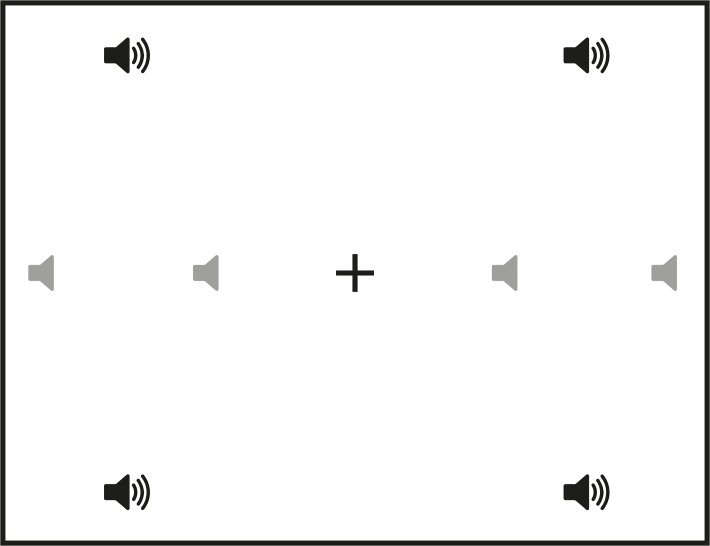
Experimental set-up. The eight speaker symbols (black and grey) depict the locations of both auditory and visual stimuli that were used in the experiment. Four of these locations (the black speaker symbols) were used for the sound selection.

Sound selection: The auditory stimuli consisted of 500 ms bursts of broadband noise. Each auditory stimulus was presented against a constant background noise (57 dB) generated by the projector. We determined for each participant the lowest intensity at which they could coarsely localize the sound stimulus in order to obtain subjectively hard-to-localize stimuli for each participant. Five sounds with a different intensity (31, 36, 41, 47 and 52 dB) were presented each 16 times randomly at 4 of the 8 used locations (Fig. 3). Participants indicated verbally in which of the four quadrants they thought the sound originated. For the experiment, the lowest intensity that was localized correctly in all trials was used. If none of the sounds were localized correctly in all trials, the highest intensity of 57 dB was used. The sound with the highest intensity had to be localized correctly in at least 75% of the trials for the patient to be included. By this criterion, six patients were excluded. Their maximum success rate varied from 38% to 69% correct localizations for one of the sounds.

The performance on the sound selection-task varied and therefore the used intensities differed. In Case 1 the used intensity was 36 dB and in Case 4 it was 52 dB. In Case 2, the sound selection-task did not work properly due to technical problems, but at least 75% of trials were localized correctly for one of the sounds. Therefore the sound with the intensity that was selected for the previous tested patient was used (36 dB). In Case 3 and 5 a sound of 57 dB was used, which they could localize correctly in 75% and 81% of trials respectively.

Experiment: The loudspeakers were located at four positions in each hemifield, four on the horizontal meridian at 8.00° or 17.82° to the left or right from a central fixation point, the other four at oblique locations, one in each quadrant at 13.01° to the left or right and 13.08° up or down from the center (Fig. 3). For comparison, in the two other studies stimuli were presented only on the horizontal meridian at eccentricities of 8° to 56°, at 16° intervals [28,29]. All visual stimuli (fixation stimulus and peripheral targets) were black (luminance of 3.79 cd/m^2^) on a grey background (luminance of 54.5 cd/m^2^) to minimize contributions of light scatter. Each trial started with a central fixation stimulus (+; 0.94° x 0.94°). After a variable period of 550 to 950 ms the auditory target was presented. On some trials a visual stimulus (disk with a diameter of 0.94°) appeared simultaneously, also for 500 ms. Participants were instructed to fixate the center stimulus until the auditory target appeared, at which time they were to move their eyes as quickly as possible to its location. It was stressed that they should ignore the visual stimulus. Each session started with a 9-point grid calibration procedure whereby patients were guided to look at the locations in their blind hemifields. Again, to ensure that participants were fixating the center of the screen at the start of each trial, the experimenter at the control monitor only started the next trial by pressing the space bar when they verified that participants were fixating screen center.

Three testing conditions were used:

1. Unimodal: the auditory stimulus was presented in isolation.
2. Bimodal coincident: the auditory stimulus was presented together with a temporally and spatially coincident visual stimulus.
3. Bimodal disparate: the auditory stimulus was paired with a temporally coincident but spatially disparate visual stimulus. Disparate visual stimuli were presented at one of the remaining three positions in the same hemifield as the location of the auditory stimulus.

The experiment consisted of a practice session of 40 trials and an experimental session of 600 trials. The following trials were presented in random order within the same block of 40 trials: 120 unimodal trials (15 for each of the 8 positions); 120 bimodal coincident trials (15 for each of the 8 positions); and 360 bimodal disparate trials (15 for each of the 24 bimodal spatially disparate conditions).

#### Data analysis

The initial saccade starting position had to be within 2° from the center fixation point in both the horizontal and vertical directions. Trials were excluded if there was no saccade, if the first saccade was < 3° in amplitude or if the endpoint of the first saccade was located in the wrong hemifield. Saccade latency was defined as the interval between target onset and the initiation of a saccadic eye movement. If saccade onset latency was either shorter than 80 ms, longer than 800 ms, or 2.5 standard deviations from the participant’s mean latency, the trial was excluded. These exclusion criteria led to a loss of 5% of trials in Case 1, 9% in Case 2, 23% in Case 3, 3% in Case 4 and 8% in Case 5.

Saccades that are captured by a distractor are in general shorter (there is an undershoot) compared to saccades that are hits [50]. Therefore we analyzed only the direction of the eye movement and ignored the absolute distance between target and saccade landing position. Saccadic accuracy was defined by calculating endpoint deviations in polar coordinates as the angular shift of the endpoint relative to the angle of the vector between the saccade starting position and the target location.

For each trial, the absolute endpoint deviation was subtracted from the mean absolute endpoint deviation in the unimodal condition for that specific target location. When this resulted in a positive value, it meant the endpoint in the bimodal condition was more accurate than the endpoint in the unimodal condition. A negative value referred to the opposite. For the calculations in the bimodal disparate condition, trials with auditory and visual stimuli that were both located on the horizontal meridian were excluded from analysis, since no angular shift would be expected.

Analyses were performed at a single-subject level. To verify that the expected distractor effects were present in the intact hemifield, saccade endpoints in both hemifields were analysed. We did not analyze whether saccades differed between the intact and blind hemifield [43] or between the coincident and disparate condition, but whether each bimodal condition differed from the unimodal condition. Per condition 10 *t*-tests were performed, with an adjusted level of significance of .005.

### Results

#### Visual field test

In Cases 1 and 2, the visual field test produced results consistent with their clinical perimetry. Cases 3 and 4 reported to see one or two stimuli respectively presented up to 8.00° horizontally in the blind hemifield, but at positions 13.08° above or below the locations used in the experiment. Therefore, the concerning fields were still considered as ‘blind’. Case 5 was only blind for stimuli at the locations in the upper right quadrant, so the lower right quadrant was not included in their analysis.

#### Saccadic accuracy

**Fig 4:**
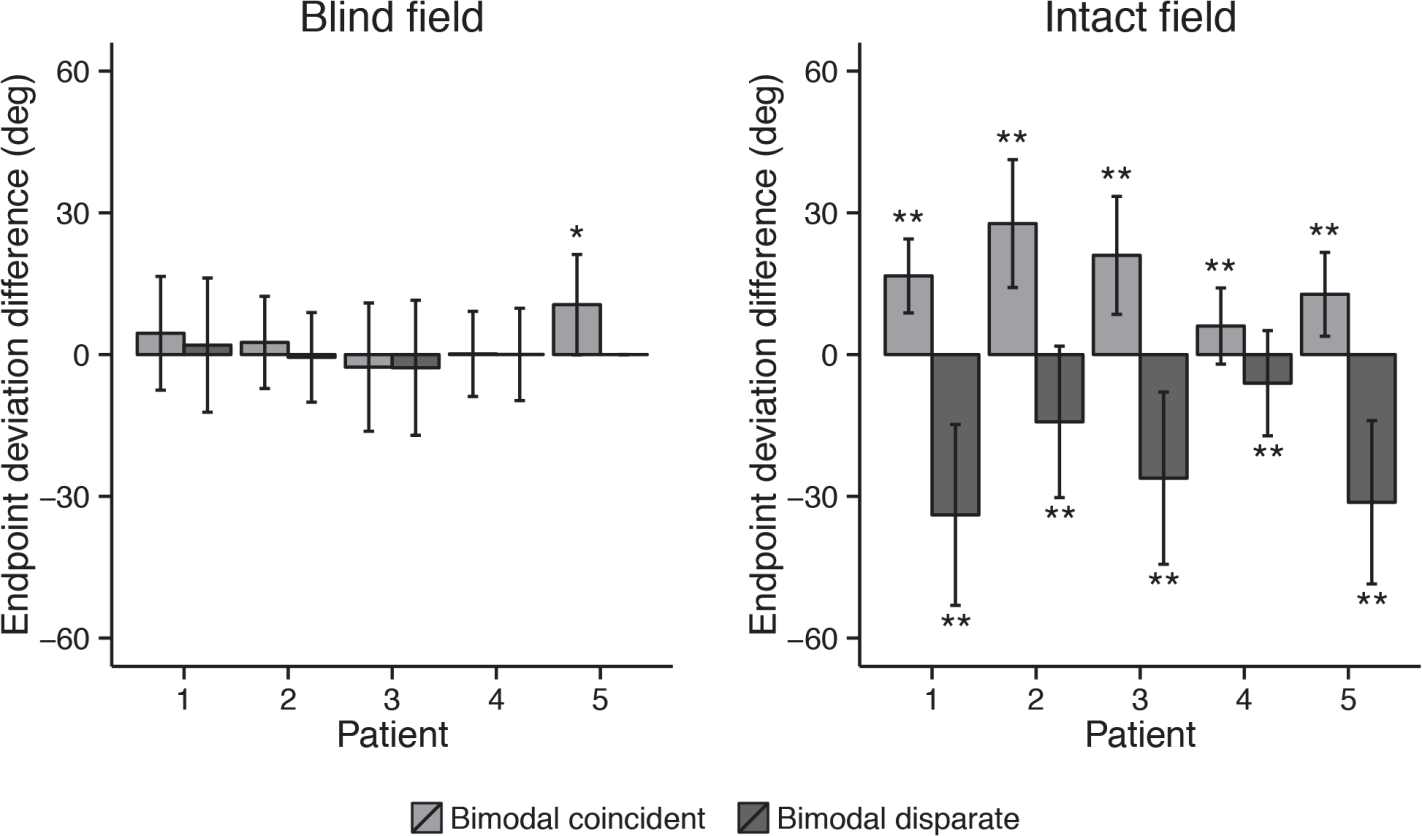
Mean endpoint deviation differences between the unimdal and bimodal conditions. Positive values indicate more accurate endpoints in the bimodal condition compared to the unimodal condition and negative values indicate less accurate endpoints compared to the unimodal condition. Error bars depict standard deviations. Asterisks indicate a significant change in endpoints (* at ɑ = .005; ** at ɑ = .001). No data is displayed for the bimodal disparate condition in the blind visual field of Case 5, since target and distractor could never be in the blind quadrant together.

The raw data of experiment 1 can be found in S1 Dataset. In the intact ipsilateral hemifield of all hemianopic patients, endpoints were significantly more accurate in the bimodal coincident condition compared to the unimodal condition. Additionally, endpoints were significantly less accurate in the bimodal disparate condition (Fig. 4; all *p* < .001). In the blind hemifield of the hemianopic patients, no effects of the visual distractor were observed (all *p* > .05). Only Case 5 showed a facilitation effect for the bimodal coincident condition in the blind quadrant (*p* = .002); note that this subject could not be tested with the bimodal disparate condition because of his more limited field loss.

#### Saccade latency

**Fig 5:**
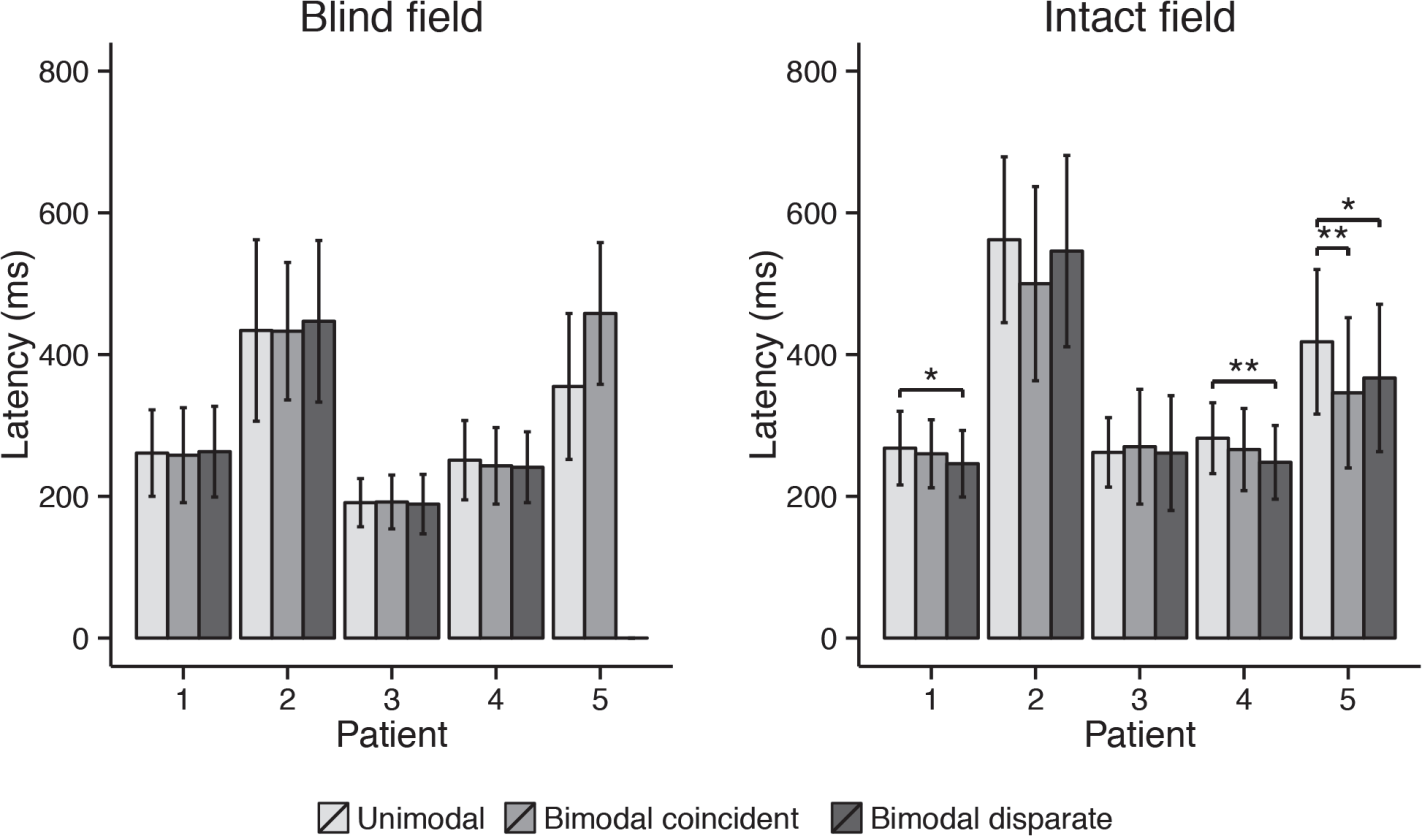
Mean saccade latency of each patient in the three different conditions. Error bars depict standard deviations. Asterisks indicate a significant change in endpoints (* at ɑ = .005; ** at ɑ = .001). No data is displayed for the bimodal disparate condition in the blind visual field of Case 5, since target and distractor could never be in the blind quadrant together.

There were effects of condition in the intact ipsilateral hemifield of some but not all hemianopic patients (Fig. 5). The latency was shorter in the bimodal disparate than in the unimodal condition for both Case 1 (*p* = .004) and Case 4 (*p* < .001), but not in the bimodal coincident condition. In Case 5, the latency was shorter in both bimodal conditions when compared to the unimodal condition (bimodal coincident: *p* < .001; bimodal disparate: *p* = .002). In the blind hemifields no differences were observed in any patient (all *p* > .05).

### Discussion

In the intact hemifield, saccadic accuracy was enhanced when the visual stimulus was spatially coincident with the auditory target and less accurate and biased towards a visual stimulus that was spatially disparate with respect to the auditory target. For the blind field, a facilitation effect was only found when the visual stimulus was spatially aligned with the auditory target in the blind visual field of the one patient with quadrantanopia. No effects of the visual stimulus were observed in the blind visual fields of the four patients with more extensive hemianopia.

Results showed shorter latencies in some bimodal conditions in the intact hemifield of some hemianopic patients, and no effect of visual stimuli in the blind field. The absence of clear effects on saccade latency in the intact visual field might be explained by the relatively large differences in saccade latency. The finding that some of the patients responded relatively slowly compared to other comparable experiments [4,33,34,41] could be due to several factors. First, faster responses may occur with more precise instructions. In this first experiment, participants were told only a few times to respond as fast as possible and were not reminded during the experiment. Second, the auditory and visual stimuli followed immediately after the moment the fixation cross disappeared. It is known that saccade latencies are reduced when the fixation cross disappears before the appearance of a peripheral target, the so called gap effect [51,52]. Regardless, the relatively high latencies might have obscured any effects of multimodal integration in these patients.

Besides the long reaction times, the setup of the experiment was perhaps inadequate to address our hypotheses. Against expectations, half of the patients could not correctly localize the sounds in the sound selection-task in more than 75% of trials, which indicates that the task might have been too challenging. Comparable tasks in other studies have revealed that people are able to localize and make eye movements to sounds in an experimental setting [41,53]. Several reasons could have made our task too difficult. First, low sound intensities were used and participants were not informed about the exact locations of the loudspeakers, to prevent a ceiling effect on saccadic accuracy. Second, in the experiment itself, many locations were used on different parts of the screen, which could have made it challenging to look at the right spot. Third, the locations of the auditory targets were varied vertically and horizontally. Previous studies have revealed that vertical sound localization is less accurate than horizontal sound localization [41]. Finally, the three different conditions (i.e. unimodal, bimodal coincident and bimodal disparate) were presented in the same block in random order. In case of a bimodal trial, the participant had to ignore the visual stimulus because it was uninformative about the location of the sound. This paradigm might therefore have been confusing for the participant, slowing down their reaction and negatively influencing saccadic accuracy.

To address these issues, we conducted a second experiment. Regarding saccade latency, instructions were more precise and a time gap was added between disappearance of the fixation cross and appearance of the stimulus. To make the task less difficult, sound intensities were higher, fewer locations were used, and participants were informed about these locations and received training to practice sound localization. Fourth, the experiment was divided into two blocks: in the first block all visual stimuli were informative (spatially aligned with the auditory target), while in the second block all visual stimuli were uninformative (spatially disparate with the auditory target).

We did not change the vertical alignment of the auditory target locations. In a prior study with healthy control subjects vertically aligned distractors (a distractor directly above or below the target) had a larger influence on saccadic accuracy compared to horizontally aligned distractors (a distractor directly left or right of the target) [53]. This can be explained by greater uncertainty about the vertical location of sounds, which causes a visual distractor to have a larger influence on the eye movement [53]. This logic could also apply for spatially coincident audio-visual stimuli. When the exact location of the auditory target is uncertain, a visual stimulus at the same location is likely to enhance saccade accuracy. Since we wanted to reduce the amount of target locations, we choose to keep the two vertically aligned locations in each hemifield. Finally, the visual stimulus had a large effect on the eye movements of the control group and in the intact ipsilateral hemifield of the patients, despite instructions to look at the location of the auditory stimulus. Possibly no effect occurred in the blind hemifield of some hemianopic patients because the visual stimulus was too salient. Following the rule of ‘inverse effectiveness’, multisensory enhancement in the superior colliculus is largest when both unimodal stimuli are weak [13,31]. Therefore, in the second experiment low and high contrast visual stimuli were used to increase to chance of observing multisensory integration.

## Experiment 2

### Materials and Methods

#### Participants

The same inclusion criteria as in the first experiment were used. The pre-experimental sound selection task was performed by six hemianopic patients. Again, only patients who were able to verbally localize sounds in at least 75% of trials were selected for the experiment. One of the six patients did not correctly localize at least 75% at the sound selection task and was excluded from the experiment.

The results of five patients were analyzed further. Two of them participated in the first experiment (Cases 1 and 4). All patients had normal or corrected-to-normal visual acuity. All participants gave informed consent according to the standards of the Declaration of Helsinki for a protocol that was approved by the institutional review boards of the hospital and the university.

#### Apparatus

**Fig 6:**
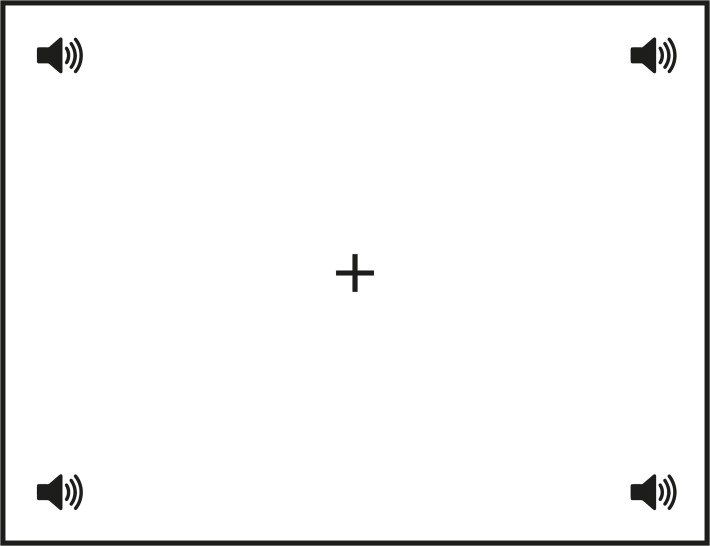
Experimental set-up. The four speaker symbols depict the locations of both auditory and visual stimuli that were used in the experiment.

In the second experiment the apparatus was the same as in the first experiment, except now four instead of eight loudspeakers were used. They were located at two positions in each hemifield, at 17.80° horizontally from a central fixation point and 13.08° up or down vertically from a central fixation point (Fig. 6). The same locations for auditory and visual stimuli were used.

#### Stimuli, procedure and design

Visual field test: The same visual field test as in the first experiment was assessed. The luminance of the background and visual stimuli were adjusted to equalize them to the stimuli which were used in the second experiment.

Training: In order to let participants practice to distinguish sounds from different locations they received a short verbal training task. The locations of the loudspeakers were clearly pointed out and the participants were told that all locations would be used during the experiment. Compared to the first experiment, five sounds with a higher intensity were used, ranging from 53 to 72 dB. They were played at the four different locations. The participant could pick the sound which he or she thought was easiest to localize. Next, this sound was played two times from each different loudspeaker whereby the location was mentioned explicitly beforehand. Finally, the location was not mentioned anymore and the participants had to guess the location of the sound whereupon feedback was provided. This procedure was repeated until the participant correctly localized four sounds in a row.

Sound selection: The same sound selection-task as in the first experiment was used in order to objectively obtain similar sounding stimuli for each participant. The four used locations were the same as those in the experimental setup of the second experiment (Fig. 4). The stimulus with the lowest intensity which was localized correctly in all trials was selected. If this was not the case for any of the sounds, the stimulus which was correctly localized in most of the trials was chosen. Participants who could not localize any sound correctly in at least 75% of the trials were excluded from the experiment. This was the case for one participant who had a maximum correct response rate of 69% of trials for one of the sounds. For the remaining participants, the used intensities were 59 dB in Case 4, 64 dB in Case 1 and 68 dB in Case 7, who could correctly localize their sound in all trials. A sound intensityof 72 dB was used in Cases 6 and 8, which they could localize correctly in 88% and 81% of trials respectively.

Experiment: The set-up of the second experiment was largely the same as the first experiment. In the second experiment the fixation stimulus and high contrast peripheral targets were black (luminance of 2.16 cd/m^2^) on a light grey background (luminance of 581 cd/m^2^). Additionally, a low contrast peripheral target was used in half of the bimodal trials (luminance of 534 cd/m^2^). Furthermore, a time gap of 100 ms was added between the fixation cross and auditory target. To improve saccadic accuracy and reduce saccade latencies, this experiment consisted of two blocks. In the first block of the experiment, solely unimodal and bimodal coincident trials were presented, whereby the second block consisted of unimodal and bimodal disparate trials. Both blocks of the experiment consisted of a practice session of 32 trials and an experimental session of 480 trials. Furthermore, the contrast of the visual stimulus was varied. In each block, 160 trials were presented for both conditions (40 for each of the 4 positions). After each trial in the practice session, feedback was given about the accuracy of the eye movement. During the whole experiment feedback was given about the speed of the eye movements, whereby participants were encouraged to respond as fast as possible.

#### Data analysis

Values for saccadic accuracy were calculated in the same manner as in the first experiment. The second experiment consisted of two separate blocks in which unimodal and bimodal trials were present. When comparisons between bimodal and unimodal conditions were made, only data from that particular block of the experiment was used.

In the first experiment, the deviation of saccades to the upper or lower targets could potentially vary up to the other (lower/upper) side of the screen, while saccades to the targets in the center of the screen could deviate only half of this distance. Therefore, in the previous experiment, we normalized each endpoint deviation in the bimodal conditions respect to each target location in the unimodal condition. This second experiment used only targets in the corners. Endpoint deviations in the bimodal trials were therefore not normalized with respect to the unimodal condition, but we averaged deviations of the different conditions instead. Per condition 10 *t*-tests were performed with an adjusted level of significance of .005.

### Results

#### Visual field test

In Cases 1 and 6, the visual field test produced results consistent with their clinical perimetry. Case 4, 7 and 8 reported to see one or two stimuli respectively presented up to 8.00° horizontally in the blind hemifield, yet these stimuli were located 13.08° above or below locations that were used in the experiment. Therefore, the concerning fields were still considered as ‘blind’.

#### Saccadic accuracy

**Fig 7:**
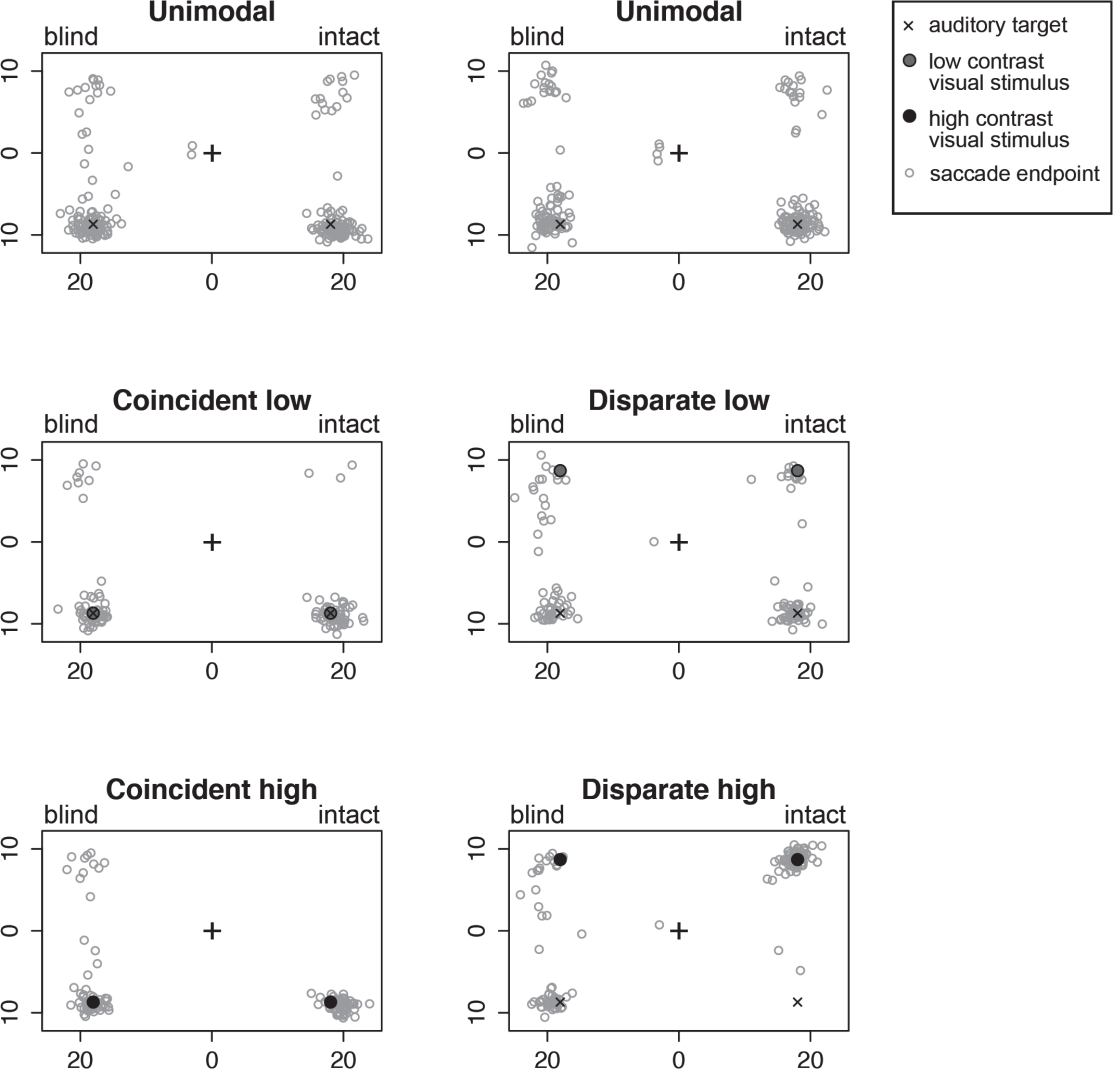
Representation of all saccade endpoints per condition for Case 1. X and y axes display horizontal and vertical position in visual degrees. Endpoint coordinates in trials with an auditory target in the upper field are flipped vertically so that all auditory targets (black ‘X’) are depicted as located in the lower field. Endpoints on the left are located in the blind hemifield, whereas endpoints on the right are located in the intact hemifield. Endpoints in the wrong hemifield are not depicted. The left three graphs show endpoints of saccades made in the blocks with bimodal coincident visual targets, whereas the right three graphs depict endpoints of saccades made in the blocks with bimodal disparate visual targets. The location of the visual stimulus is depicted as a grey (low contrast) or black (high contrast) filled circle.

Bimodal coincident condition: The raw data of experiment 2 can be found in S2 Dataset. Fig. 7 plots the saccade endpoints for Case 1 as an example. Endpoints are plotted separately for the unimodal condition, the coincident low contrast condition, and the coincident high contrast condition. Endpoint coordinates in trials with an auditory target in the upper field are flipped vertically so that all auditory targets are depicted as located in the lower field. In all graphs, endpoints in the left half were made to a target in the blind hemifield and endpoints in the right half were made to a target in the intact hemifield. We depicted only trials that were included in the analyses. For example, trials with endpoints that were located in the opposite half relative to the target are not depicted.

**Fig 8:**
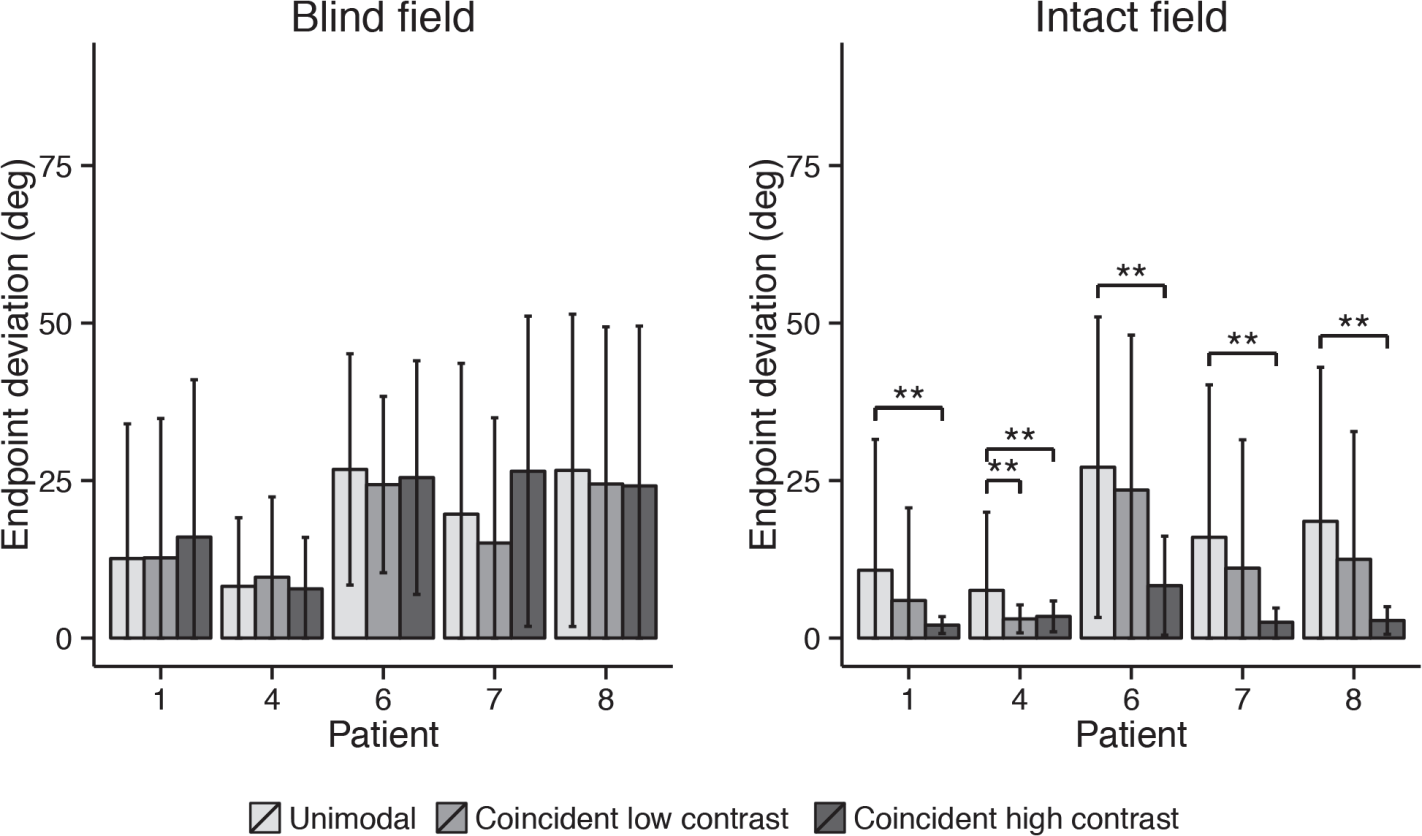
Mean endpoint deviations of each patient in the three different conditions of the first block (coincident) of the experiment. Error bars depict standard deviations. Asterisks indicate significant pairwise comparison between unimodal and bimodal conditions (* at ɑ = .005; ** at ɑ = .001).

In the intact ipsilateral hemifield of all subjects, a high contrast coincident visual stimulus made saccades more accurate than those in the unimodal condition (Fig. 8; all *p* < .001). When a low contrast coincident visual stimulus was used, this improved saccadic accuracy in the intact hemifield of only Case 4 (*p* < .001). Neither low nor high contrast coincident visual stimuli had any effect on saccade endpoints in the blind hemifield of the patients (all *p* > .05).

Bimodal disparate condition: In the second block of the experiment, the visual stimulus was always projected at a different location than the auditory target in case of a bimodal trial. Fig. 7 illustrates the results for Case 1 as an example.

**Fig 9:**
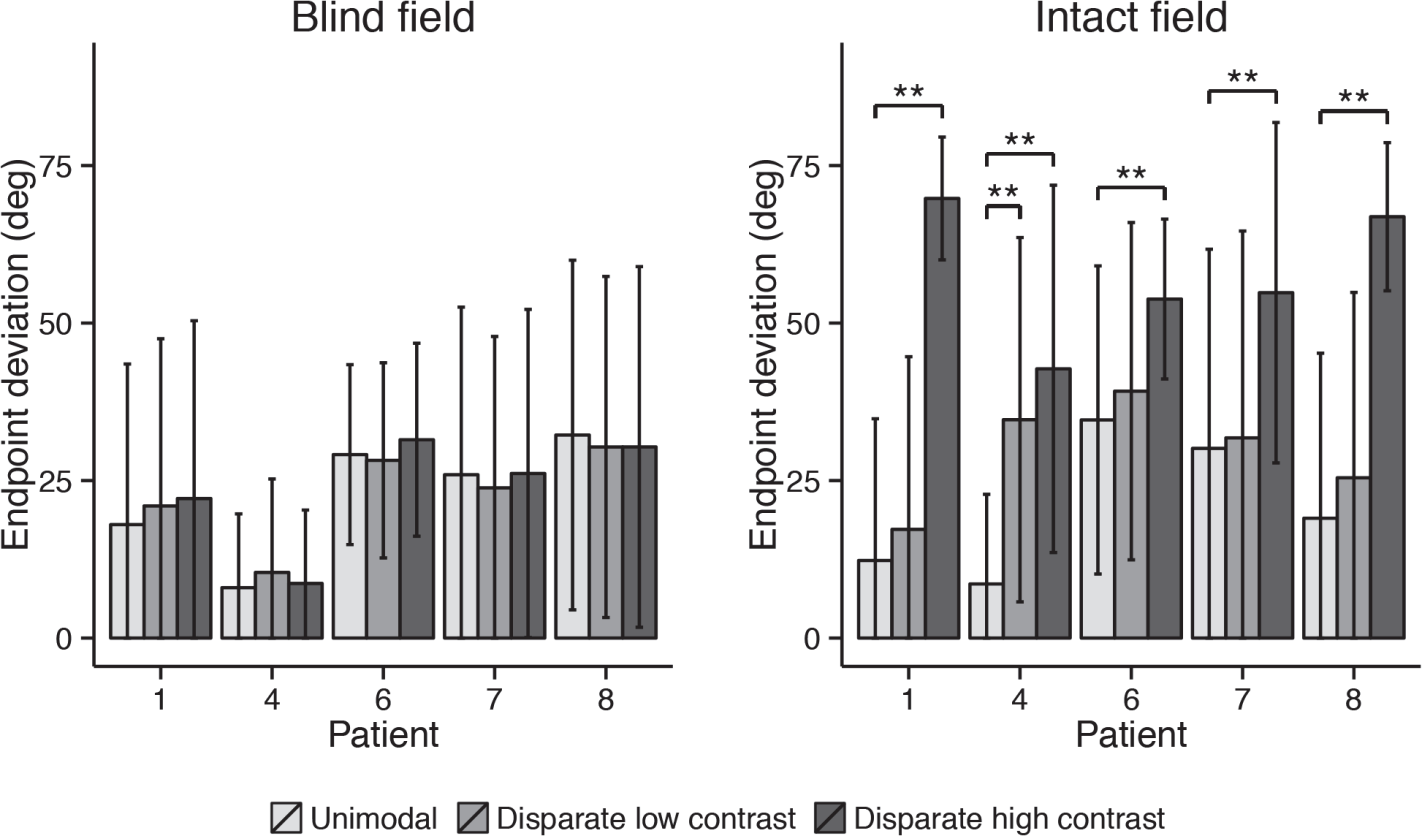
Mean endpoint deviations in the three different conditions of the second block (disparate) of the experiment. Error bars depict standard deviations. Asterisks indicate significant pairwise comparison between unimodal and bimodal conditions (* at ɑ = .005; ** at ɑ = .001).

The mean saccade endpoint deviations are depicted in Fig. 9. The high contrast disparate visual stimulus caused endpoints to deviate away from the auditory target in the intact ipsilateral hemifield of all patients (all *p* < .001). The low contrast disparate visual stimulus affected the saccades in the intact hemifield of only Case 4 (p < .001). Again, neither one of the two disparate visual stimuli had any effect on saccade endpoint in the blind hemifield of the patients (all *p* > .05).

#### Saccade latency

**Fig 10:**
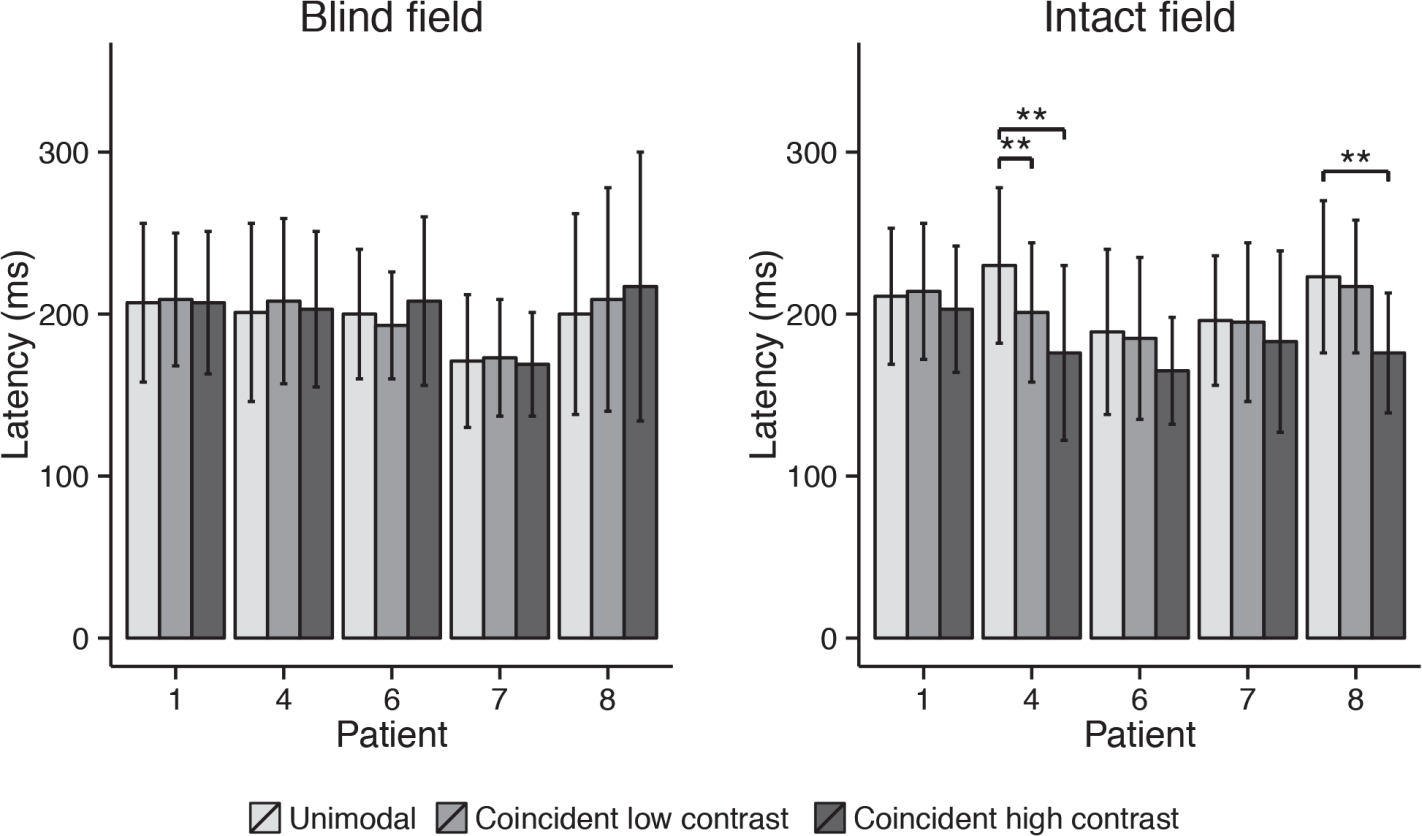
Mean saccade latency for each patient in the three different conditions of the first block (coincident) of the experiment. Error bars depict standard deviations. Asterisks indicate significant pairwise comparison between unimodal and bimodal conditions (* at ɑ = .005; ** at ɑ = .001).

Bimodal coincident condition: In the intact hemifield saccade latencies tended to be shorter in the high contrast coincident trials compared to unimodal trials (Fig. 10), but this difference was only significant for Cases 4 and 8 (*p* < .001). There was a trend for Case 6 (*p* = .012). The low contrast coincident visual stimulus affected only the mean latency of Case 4 (*p* < .001). In the blind hemifield, neither low nor high contrast coincident visual stimuli affected the saccade latency of the participants in de first block of the experiment (all *p* > .05).

**Fig 11:**
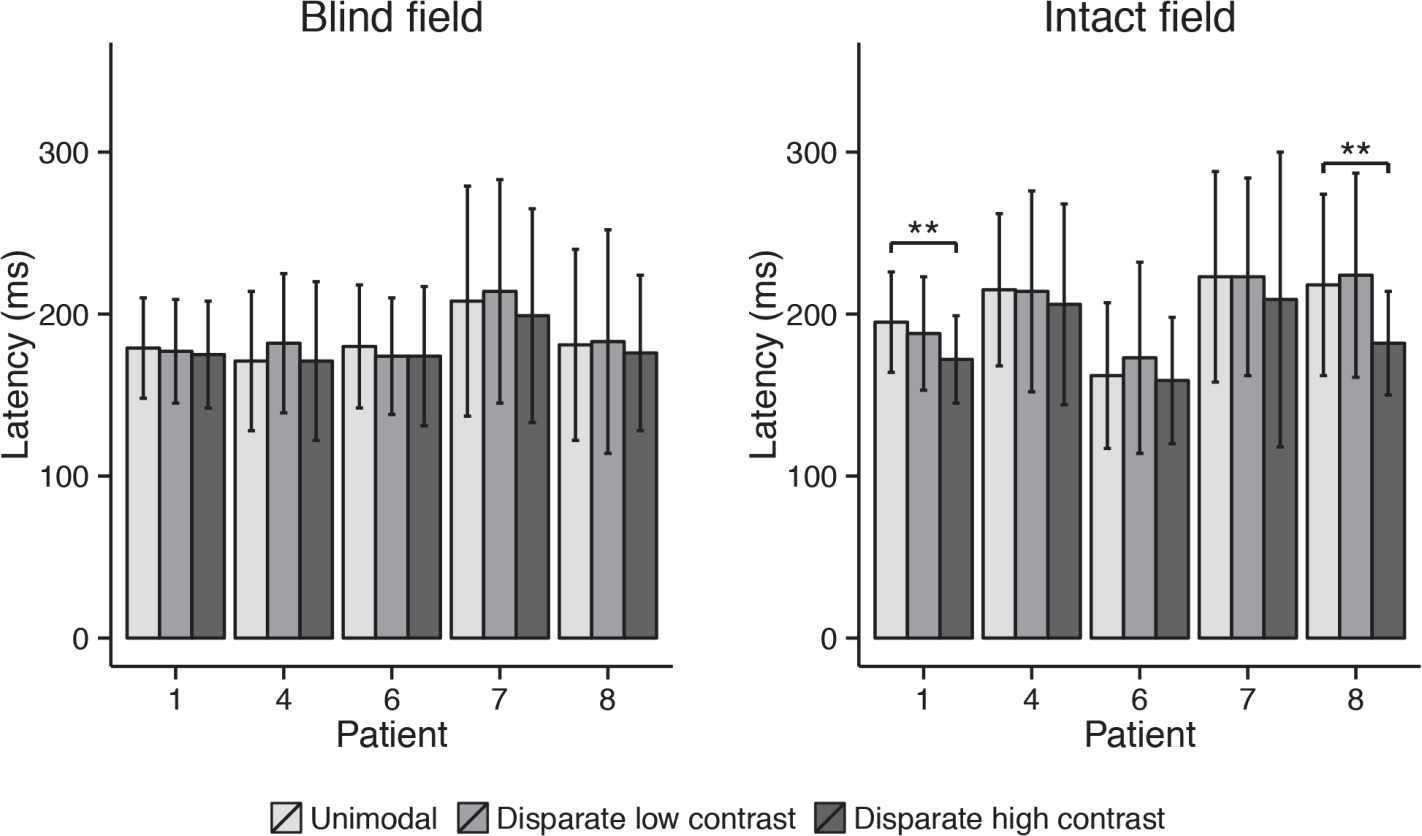
Mean saccade latency for each patient in the three different conditions of the second block (disparate) of the experiment. Error bars depict standard deviations. Asterisks indicate significant pairwise comparison between unimodal and bimodal conditions (* at ɑ = .005; ** at ɑ = .001).

Bimodal disparate condition: Saccade latencies were significantly shorter when a high contrast disparate visual distractor was presented in the intact ipsilateral hemifield for Cases 1 and 8 (*p* < .001). The low contrast disparate visual stimulus seemed to have no effect (Fig. 11; all *p* > .05). In the blind hemifield, neither of the visual stimuli did affect saccade latency of the participants (all *p* > .05).

## Discussion

We investigated whether a visual stimulus in the blind hemifield influences saccades made to an auditory stimulus. In neither of the two experiments did we observe an effect of the visual stimulus in the blind hemifield of the seven hemianopic patients, although in both experiments there was a clear effect of the visual stimulus on saccades to the intact hemifield. Only in the patient with quadrantanopia was there a facilitation effect in the blind quadrant.

The finding that visual stimuli influenced saccade accuracy strongly in the intact visual field of all patients indicates that the null effect in the blind visual field is not due to methodological issues. However, whether signs of blindsight can be observed in a given patient seems to depend largely on the method of testing [54]. Multiple forms of blindsight have been distinguished, e.g. attention-blindsight reflects residual abilities such as detecting or discrimination of stimuli in the blind field, whereas in action-blindsight patients are able to accurately act towards blind field stimuli, such as pointing or making saccades to them [43,55]. These forms are closely related, however, and the distinction between them is not always evident. The implicit processing used in our study, in which the effect of blind stimuli on responses to perceived stimuli is investigated, is sometimes labelled as assessing action-blindsight [9] and sometimes as assessing attentional-blindsight [55]. Prior studies that showed multisensory integration at group level in the blind field of hemianopic patients used experimental paradigms that involved reports of presence [28] or location [29] of visual stimuli, which is more clearly related to attentional-blindsight. Frassinetti et al. [28] asked hemianopic patients to verbally report presence of a visual stimulus that was presented in the blind hemifield. An LED light was either presented alone or combined with an auditory stimulus that was spatially coincident or disparate with the target. They observed that conscious awareness of a blind-field visual stimulus in seven hemianopic patients was enhanced when it was spatially coincident with an auditory stimulus, whereas we studied how saccadic responses to an auditory stimulus were altered by a visual stimulus in the blind field. Although we assumed that eye movements would be more sensitive as a measure of multimodal integration, no effects were seen. However, the fact that Frassinetti et al.’s [28] patients responded on 4–9% of trials with visual targets alone indicate either a baseline rate of guessing or some residual vision. Guessing seems less plausible given that only on 0–2% of auditory-alone catch trials did patients claim to see a visual stimulus.

Another methodological difference is that we used both horizontal and oblique locations, in contrast to previous studies that presented only distractors on the horizontal meridian [28,29]. Other work has shown that vertical sound localization is less accurate compared to horizontal sound localization [41], and that vertically aligned visual distractors have a greater influence on eye movements compared to horizontally aligned distractors [53]. Although we hypothesized that vertical distractors would result in stronger effects than in prior studies, no effects were found for stimuli in the blind hemifield of hemianopic patients. As in the study of Leo et al. [29] we attempted to obtain hard-to-localize stimuli, in order to prevent a ceiling effect of accurate localizations to occur. One might argue that the lack of any effect of the visual stimuli on saccadic eye movements in the blind field of the hemianopic patients could be due to insufficient localization accuracy of the auditory stimulus in the vertical plane. However, the participants included in our experiments could accurately localize the sounds in the unimodal condition. Furthermore, in the patient with quadrantanopia a facilitation effect of the visual stimulus was seen when it was aligned with the auditory target. This indicates that the visual stimulus could affect eye movements made in the blind visual field, due to multisensory integration, although underlying mechanisms in quadrantanopia might not be comparable with those in hemianopia.

The observation that an influence of the visual stimulus in the intact hemifield could not be found in the blind hemifield emphasizes that experimental parameters can be critical in blindsight. For example, in a case study of Carey et al. [56] a dissociation was found between manual and saccadic localisation when they used targets with both vertical and horizontal displacements. Only manual localisation in the blind field could be demonstrated, whereas saccadic localisation was impaired by adding the vertical dimension. The interactions between localisation type (saccadic or manual), stimulus location (vertical or horizontal alignment) and other aspects of the experimental design (attention- or action-blindsight, implicit or explicit measurements) in blindsight are not yet fully understood.

Evidently, not all patients with a lesion of the optic radiations or striate cortex show residual visual functions in the blind hemifield. In studies that examined groups of hemianopic patients, only a few cases showed blindsight [4,6–8]. This could be due to individual differences regarding specific brain injury and post-traumatic functional recovery. There could be residual visual functions in the primary pathway [10] or more than one single alternative pathway could be mediating different expressions of blindsight, which strengths could change over time due to plasticity [9,57]. Effects of structural and functional heterogeneity may impact studies with a small number of patients. The effects of functional differences remains unclear, given continued debates about the substrate of blindsight [10,54,57].

To summarize, multimodal interactions were observed in the blind hemifield of none of the seven patients with hemianopia, though a facilitatory effect of the visual stimulus was observed in one patient with quadrantanopia. Although this indicates that multimodal stimuli can influence the oculomotor competition in patients with visual field defects, the results are not convincing and do not confirm that the SC plays a role in blindsight [1,11–16]. The fact that blind-field effects are not found in all patients with only cerebral damage and no evident lesion that would disrupt a retinotectal pathway fit with the idea that the effects of multimodal integration in the blind visual field are not based on a general mechanism.

## Supporting Information

S1 Dataset Dataset of experiment 1. (ZIP)

S2 Dataset Dataset of experiment 2. (ZIP)

S1 Explanation Data Explanation of data files. (DOC)
