# Supplementary material for: Lack of Multisensory Integration in Hemianopia: No Influence of Visual Stimuli on Aurally Guided Saccades to the Blind Hemifield
Source: PLoS One. 2015 Apr 2;10(4):e0122054. doi: 10.1371/journal.pone.0122054 (PMC4383622; doi:10.1371/journal.pone.0122054)
Supplement: S1 Explanation Data — (DOC) [file pone.0122054.s003.doc]

**Data - Lack of multisensory integration in hemianopia: no influence of visual stimuli on aurally guided saccades to the blind hemifield**

For each patient who was included in the analyses:

**Experiment 1**

- …EXP.ma: raw data

Columns include:

- - Trial number 0-39 training, 40-639 experiment
  - Auditory target location 0=left, 1=up, 2=right, 3=down
  - Visual stimulus location 0=none, 1=left, 2=up, 3=right, 4=down
  - Side 0=left, 1=right
  - Visual stimulus x-coordinate
  - Visual stimulus y-coordinate
  - Latency
  - Saccade start position x-coordinate
  - Saccade start position y-coordinate
  - Saccade end position x-coordinate
  - Saccade end position y-coordinate


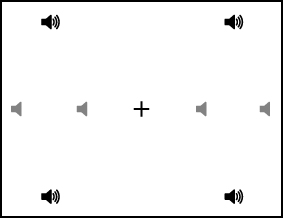


**Figure 3.** The eight speaker symbols (black and grey) depict the locations of both auditory and visual stimuli that were used in experiment 1.

- …accuracy_SPSS: contains all included trials (trials are excluded based on criteria stated in the manuscript)

Columns include deviation differences between unimodal and bimodal conditions:

- - left_deviation_difference_coincident
  - left_deviation_difference_disparate
  - right_deviation_difference_coincident
  - right__deviation_difference_disparate
- …latency_SPSS: contains all included trials (trials are excluded based on criteria stated in the manuscript)

Columns include:

- - Condition LEFT: 1= unimodal, 2=coincident, 3=disparate

RIGHT: 4= unimodal, 5= coincident, 6= disparate

- - Latency

**Experiment 2**

- … EC1.ma and …EC2.ma (coincident block) and ED1.ma and ED2.ma (disparate block).

Columns include:

- - Trial number file1: 0-31 training, 32-255 experiment

file2: 0-255 experiment

- - Auditory target location 0=left up, 1=left down, 2=right up, 3=right down
  - Contrast 0=low, 1=high
  - Condition 0=unimodal, 1=bimodal
  - Visual stimulus X-coordinate
  - Visual stimulus y-coordinate
  - Latency
  - Saccade start position x-coordinate
  - Saccade start position y-coordinate
  - Saccade end position x-coordinate
  - Saccade end position y-coordinate
- …SPSS: contains all included trials (trials are excluded based on criteria stated in the manuscript).

Columns 1-3 contain data from the coincident block, columns 4-6 contain data from the disparate block.

- - Condition LEFT: 1= unimodal, 2=bimodal low, 3=bimodal high

RIGHT: 4= unimodal, 5=bimodal low, 6=bimodal high

- - Latency
  - TSEabs angle between target, saccade start position and saccade end position
